# Position of the AI for Health Imaging (AI4HI) network on metadata models for imaging biobanks

**DOI:** 10.1186/s41747-022-00281-1

**Published:** 2022-07-01

**Authors:** Haridimos Kondylakis, Esther Ciarrocchi, Leonor Cerda-Alberich, Ioanna Chouvarda, Lauren A. Fromont, Jose Manuel Garcia-Aznar, Varvara Kalokyri, Alexandra Kosvyra, Dawn Walker, Guang Yang, Emanuele Neri, the AI4HealthImaging Working Group on metadata models**

**Affiliations:** 1 FORTH-ICS, Heraklion, Greece; 2 Department of Translational Research, University of Pisa, Pisa, Italy; 3 Biomedical Imaging Research Group, La Fe Health Research Institute, Valencia, Spain; 4 Aristotle University of Thessaloniki, Thessaloniki, Greece; 5 Centre for Genomic Regulation (CRG), The Barcelona Institute of Science and Technology, Barcelona, Spain; 6 University of Zaragoza, Zaragoza, Spain; 7 Department of Computer Science and Insigneo Institute of in silico Medicine, University of Sheffield, Sheffield, UK; 8 National Heart and Lung Institute, Imperial College London, London, UK

**Keywords:** Artificial intelligence, Diagnostic imaging, Metadata, Radiomics, Radiation therapy

## Abstract

A huge amount of imaging data is becoming available worldwide and an incredible range of possible improvements can be provided by artificial intelligence algorithms in clinical care for diagnosis and decision support. In this context, it has become essential to properly manage and handle these medical images and to define which metadata have to be considered, in order for the images to provide their full potential. Metadata are additional data associated with the images, which provide a complete description of the image acquisition, curation, analysis, and of the relevant clinical variables associated with the images. Currently, several data models are available to describe one or more subcategories of metadata, but a unique, common, and standard data model capable of fully representing the heterogeneity of medical metadata has not been yet developed. This paper reports the state of the art on metadata models for medical imaging, the current limitations and further developments, and describes the strategy adopted by the Horizon 2020 “AI for Health Imaging” projects, which are all dedicated to the creation of imaging biobanks.

## Key points

- Metadata are essential for the correct use and interpretation of medical images.
- An appropriate and possibly standardised data model is necessary to represent these data and their correlations.
- We report the state of the art of metadata models and the position of Horizon 2020 “AI for Health Imaging” projects.

## Background

Metadata, as the word suggests, are data about the data, *i.e*., additional information about the data themselves. For medical imaging, these include data generated from an imaging modality, exam prescription codes, description data based on an order, and annotations indicating the content and/or anatomy of a particular image [1]. Other essential metadata are imaging biomarkers and clinical variables, *i.e*., complementary non-imaging data related to the patient’s medical history that are necessary for a correct diagnosis and decision.

In order to efficiently use the medical data, it is crucial to properly combine the actual imaging data with their associated metadata [2]. For this, the appropriate models need to be available to enable homogeneous data access and analysis. Another aspect worth mentioning is that, depending on the specific imaging biobank and its focus, the metadata to be collected may vary, as it will be shown later on in this paper. Up to date, several models exist that allow to describe and sometimes standardise one or more subdomains of medical imaging metadata. What is still missing is the definition of a unified, complete, and standardised model that is able to fully represent this new type of metadata.

To this purpose, this paper provides an overview on the metadata for medical imaging, on the currently available dedicated models and their problems and limitations, and presents the modeling strategy adopted by the Horizon 2020 Artificial Intelligence In Health Imaging (AI4HI) Network, comprising the PRIMAGE [3, 4], EuCanImage [5], CHAIMELEON [6, 7], INCISIVE [8], and ProCancer-I [9] Horizon 2020 projects.

### State-of-the-art of metadata models for medical imaging

In this section, we describe in detail what are the relevant metadata for an imaging biobank, and we report on the current approaches available to establish common metadata models for medical imaging. In the field of databases, a data model is an abstract scheme that organises the data, their properties, and how they are related to one another. As it will be shown, for medical images currently there are several available models, which are able to represent a subset of the metadata contained in an imaging biobank with different levels of accuracy. What is missing so far is a unification and a standardisation of these models, *i.e*., one common and comprehensive model able to fully describe the content on an imaging biobank and properly link the different domains (*e.g*., images, clinical variables, radiomics).

#### Metadata in imaging biobanks

##### Imaging data and clinical data

**Fig. 1:**
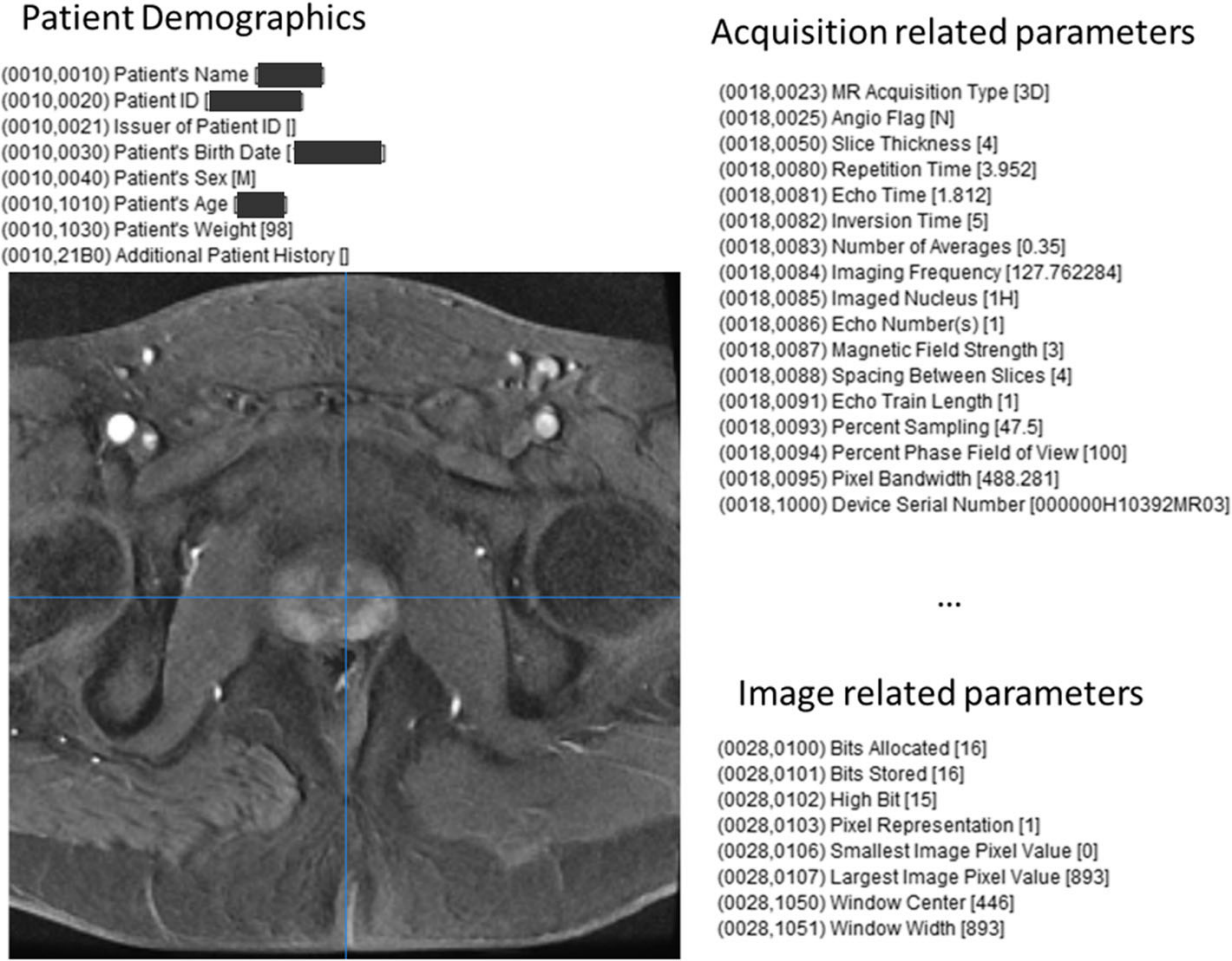
An example of a Digital Imaging and Communications in Medicine (DICOM) contrast-enhanced magnetic resonance image (T1-weighted sequence) of the prostate and DICOM metadata about patient demographics, acquisition-related parameters and image-related parameters

The core of metadata in medical imaging is represented by the Digital Imaging and Communications in Medicine (DICOM) standard that defines the acquisition, the exchange, and the processing of images and associated metadata in the medical domain. The DICOM file includes metadata that describe the patient's demographic, the modality and acquisition parameters, and other imaging-related parameters. An example is shown in Fig. 1, presenting a DICOM magnetic resonance (MR) image of the prostate and the relevant DICOM metadata describing patient demographics, acquisition, and image-related parameters.

Other imaging metadata might also include radiology reports, which can be in a free-text format or a structured format (structured reporting). A radiology report is generated by the human interpretation of images associated with clinical data and contains intrinsic information that can be transformed into metadata. Many natural language processing applications to extract quantitative information from free-text reports are already available [10]. However, initiatives promoted by national and international radiological scientific societies (*e.g.*, the Radiological Society of North America [11] and the European Society of Radiology (ESR) [12]) are speeding up the adoption of structured reports in clinical practice. In fact, structured reports already have the appropriate format and content for metadata extraction, and therefore represent an important source of metadata for large-scale analysis of patient cohorts, for artificial intelligence training and multiscale simulations, which can be developed incorporating mechanistic biological and physical processes at the scale of the protein, cell, tissue, and organ. The models can generate predictions such as tumour growth or shrinkage under specific chemotherapeutic treatment, which can then be validated against image-based data.

#### Imaging biomarkers

Imaging biomarkers can be considered as well as a subset of metadata, objectively measured and evaluated as an indicator of normal biological processes, pathogenic processes, or biological responses to therapeutic interventions [13]. Imaging biomarkers can be either quantitative (*e.g*., lesion diameter or volume, computed tomography-based density, MR signal intensity, radiomics features, and any other biomarker whose magnitude can be expressed as a quantity value) or qualitative (*e.g.*, pathological grading systems that can be expressed as ordinal rather than continuous quantitative data, such as clinical TNM staging, diagnostic categories defined according to “reporting and data systems” such as BI-RADS, LI-RADS, PI-RADS, C-RADS, etc.) [14, 15]. A number of international initiatives have been launched to promote the development and clinical implementation of image biomarkers. In 2007, the Radiological Society of North America organised the Quantitative Imaging Biomarkers Alliance®, with the aim of promoting collaboration between researchers and industry players. Quantitative Imaging Biomarkers Alliance initiatives include collaborating to identify needs, barriers and solutions to the creation of quantitative biomarkers, and accelerating the development of hardware and software to obtain accurate and reproducible quantitative biomarkers. In addition, the ESR has set up a subcommittee called European Imaging Biomarkers Alliance (EIBALL), aimed at coordinating all the ESR activities related to image biomarkers. EIBALL recently provided recommendations and examples of biomarkers validated and used in clinical practice [16]. Finally, the Image Biomarker Standardization Initiative (IBSI) is an independent international collaboration dedicated to standardising the extraction of image biomarkers from images to perform quantitative image analysis (radiomics) [17].

#### Link between metadata domains

As imaging biomarkers express biological phenomena, they can be considered the imaging phenotypes of such processes, and therefore it is reasonable to search for a link/correlation between imaging and non-imaging metadata. The rationale of this link has distant origins in what is defined as radiological-pathological correlation, where the histopathological type of a tissue or a biological process has a counterpart in the visual radiological semeiotics of the radiologist. Anyway, this is a phenotype-phenotype correlation. Modern imaging is evolving from visual (subjective image-based) interpretation to quantitative interpretation, based on quantitative imaging biomarkers that express biological, pathological processes and the response of pathology to treatment. In this transformation, the quantitative biomarkers of the images become metadata that can be correlated with the metadata of other “omics” sciences, such as genomics, proteomics, etc., and are therefore at the basis of the so-called genotype-phenotype correlation [18]. As an extension to this, such diverse metadata and associated data might be key to informing computational simulation models, as is the case of the PRIMAGE project (see below). Given the importance of this link, in 2016 the ESR set up a specific DICOM-Minimum Information About BIobank data Sharing (DICOM-MIABIS) working group with the goal of linking non-imaging to imaging data [19]. Of note, imaging biomarkers can be simple biomarkers, such as lesion diameter, or more complicated biomarkers, such as the grey level co-occurrence matrix (GLCM), which is one of the most commonly used texture features in radiomics. However, these biomarkers not only express biological properties, but are also influenced by the technical settings, such as scanning protocols, this being the main reason underlying the necessity of data harmonisation.

#### Currently available metadata models

#### DICOM extensions to the clinical domains

Many working groups have been established by the DICOM Standard Committee to develop standards for a particular modality, clinical domain, or technical area. To date, the DICOM standard has set up 34 working groups, which include the radiology modalities and other non-radiological domains as imaging in dentistry, dermatology, pathology. For example, the dermatology working group aims to develop supplements to the DICOM standard for dermoscopy, total body photography, and reflectance confocal microscopy imaging [20–22].

#### Semantic DICOM

Another model that focuses on providing a metadata model on top of the DICOM images is the Semantic DICOM (SEDI) [23]. The objective of SEDI is “to support the real-time translation of semantic queries into DICOM queries” while targeting radiotherapy PACS. In this direction, data using the SEDI ontology are added to the DICOM file as metadata. Those metadata can then be stored and searched effectively using semantic web technologies. SEDI enables search through a structured query language, such as the SPARQL Protocol and Resource Description Framework (RDF) Query Language, over data available in DICOM files. The ontology offers a rich set of terms, but it has not been updated since 2015 [24].

#### MIABIS

The Minimum Information About BIobank data Sharing (MIABIS) was initiated in 2012 [25], as a recommendation about what information should be stored in biobanks to facilitate the exchange of sample information and data. The MIABIS Core version 2.0 was developed in 2016 [26] and is currently used in several biobank registers and catalogs. In the MIABIS Core 2.0, three main entities are identified, namely “Biobank,” “Sample Collection,” and “Study,” and a minimum number of attributes for each entity is reported. In 2020, three new modules called “Sample,” “Sample donor,” and “Event” were added to the MIABIS Core to describe samples and sample donors at an individual level [27]. The “Event” module, in particular, seems relevant also for imaging data, as it allows reporting of events such as a disease diagnosis or death. What is missing so far in MIABIS is the link/extension to DICOM imaging data and associated metadata. To this aim, a DICOM-MIABIS linking model has been proposed in a recent paper, as an extension of the MIABIS core, mostly with DICOM metadata [28]. The proposed conceptual model is based on the three-module original MIABIS Core 2.0, and suggests replacing the “Sample Collection” module with a more general one called “Sample”. This “Sample” module is linked on the one side to the rest of the MIABIS Core for tissue metadata, and on the other side to newly added modules, specific for images. These modules not only report a minimal set of DICOM metadata, describing heterogeneous information across datasets, such as imaging protocols, modalities, sequences, scanners, and labels, but also additional information regarding the image processing and analysis for radiomic feature extraction.

#### Observational Medical Outcomes Partnership (OMOP) Common Data Model (CDM)

The OMOP CDM allows for the systematic analysis of different and heterogeneous observational databases. Its approach is to transform data contained within the source databases into a common format (data model) with a common representation (terminologies, vocabularies, coding schemes), and then perform systematic analyses using standard methods based on the common format [29]. Just recently, an Oncology Extension [30] and a Radiology Extension [31] for the OMOP CDM have been proposed. The work is still ongoing, but the extensions will allow to represent and standardise concepts and procedures that are specific to the fields of oncology and radiology.

#### HL7 FHIR and OHDSI OMOP alliance

Health Level Seven International (HL7) and Observational Health Data Sciences and Informatics (OHDSI) have recently announced a collaboration to provide a single Common Data Model for Sharing Information in Clinical Care and Observational Research, which will address the sharing and tracking of data in the healthcare and research industries [32]. This will be done by aligning and integrating HL7 Fast Healthcare Interoperability Resources (FHIR) and OHDSI’s OMOP CDM, allowing clinicians as well as researchers to pull data from multiple sources and compile it in the same structure without degradation of the information, benefiting from the analytics and predictive modeling capabilities of OMOP and the information retrieving from FHIR due to its patient-level processes orientation. Extensive work has been done on this issue prior to the agreement, generating projects with a certain maturity on which the first designs of the collaboration could be based. One of the most relevant examples of this work is the OMOP on FHIR initiative [33]. OMOP on FHIR is an open-source platform that provides bidirectional mapping processes between OMOP CDM and FHIR. It also allows turning any data analytics process into a service (“analytics-as-a-service”) for delivery at the point of care. It acts as an FHIR wrapper for an OMOP database using a data converter (backbone) between OMOP and FHIR.

#### ICGC-ARGO

The International Cancer Genome Consortium-Accelerate Research in Genomic Oncology ICG-ARGO Data Dictionary expresses the details of a cancer-focused data model and describes the attributes and permissible values for all of the fields within the model [34]. It is used to analyse data in the ICGC platform, which contains specimens from 100,000 cancer patients with high-quality clinical data. In addition, several funded projects are using the model, including EuCanCan, an Horizon 2020 project in cancer research in Canada and Europe.

Besides the aforementioned data models, whose main features are summarised in Table 1, it is worth mentioning the possibility of adopting a model based on *common data elements* (CDEs). A CDE describes a specific data item, its specific attributes, and all their possible values according to a vocabulary that is both human and machine-readable. The use of CDEs has already been adopted in several medical fields, including radiology, where a CDE model has been defined based on existing international standards [35]. In addition, several ontologies have also been developed specifically for capturing medical imaging metadata, and, although they have not been used by the projects presented in the sequel, we mention them for reasons of completeness. First, the *Radiomics Ontology* [36] models the radiomics feature domain, includes computational details, and has tools that generate template tables for standardised reporting and scripts/tools for publishing the modeled data as linked open data. Second, the *Radiation Oncology Ontology* [37] aims to cover the radiation oncology domain, including cancer diseases, cancer-staging systems, and oncology treatments, with a strong focus on reusing existing ontologies. Third, the *Ontology-guided radiomics analysis workflow* [38] is an open-source software package that deploys a standard lexicon to uniquely describe radiomics features in common usage, and it provides methods to publish radiomic features as a semantically interoperable data graph object complying with Findability, Accessibility, Interoperability, and Reuse (FAIR) data principles, using metalabels attached from the Radiation Oncology Ontology and the IBSI compliant Radiomics Ontology. Fourth, the *Dependency Layered Ontology for Radiation Oncology* [39] was built in an effort to capture the knowledge in radiation oncology, including the dependency semantics among the identified terms. The ontology reuses other standard ontologies and terminologies, such as the International Classification of Disease 10 from the World Health Organization [40], the National Cancer Institute Thesaurus [41], the Systematized Nomenclature of Medicine Clinical Terms (SNOMED-CT) [42], and MOSAIQ oncology information system [43], and is exploited as input for building Bayesian networks for the domain of radiation oncology. Finally, *Radiation Oncology Structures Ontology* [44] describes commonly contoured (anatomical and treatment planning) structures for radiation treatment planning. It includes more than 22,000 structure labels (created over a 16-year period in a radiation department) which were extracted, classified and categorised to produce this ontology. This ontology was created to ease and standardise the integration of radiation oncology data into clinical data warehouses for multicentric studies. As stated in Ref. [44], the ontology is aligned to external ontologies like the Model of Anatomy [45] and Unified Medical Language System /SNOMED-CT [42].

**Table 1:** Summary of the most relevant metadata models currently available, the type of metadata they represent, and the scope of the model

Model	Type of metadata	Scope
DICOM extensions	Clinical variables	Extend DICOM metadata to other domains
SEDI	DICOM tags	Enable semantic search over DICOM tags
MIABIS	Biological samples and tissues	Standard for traditional biobanks and Biobanking and Biomolecular Resources Research Infrastructure–European Research Infrastructure Consortium Directory
OMOP CDM	Clinical variables	Standardise observational medical outcomes
FHIR	Clinical variables	Standard for health care data exchange
OMOP on FHIR	Clinical variables	Bidirectional mapping
ICGC-ARGO	Cancer-focused clinical variables	Standardise variables, attributes, and permissive values in the cancer domain

*DICOM* Digital Imaging and Communications in Medicine, *FHIR* Fast Healthcare Interoperability Resources, *ICGC-ARGO* International Cancer Genome Consortium-Accelerating Research in Genomic Oncology, *MIABIS* Minimum Information About BIobank data Sharing, *OMOP CDM* Observational Medical Outcomes Partnership Common Data Model, *SEDI* Semantic DICOM

#### Problems with existing metadata models and approaches

Although in the previous section we presented multiple approaches and metadata models developed for medical imaging, still many problems are left unsolved. In this section, we elaborate on the problems with existing metadata models and the corresponding approaches focusing on three key dimensions, *i.e*., the diversity of the available data to be modeled, the diversity of existing models, and the diversity of homogenisation efforts that further complicate the selection of the appropriate workflow.

##### Diversity of data

The data generated by different disciplines (such as genomic, metabolomic, proteomic, radiomics) have differences in format and structure that make correlation difficult. The problem of correlation between data of different nature is certainly in the domain of statistics, which provides various tools and solutions. At the same time, the large amount of data available from the different omics sciences requires a high computational capacity for their efficient correlation [46]. It is therefore necessary to group the data by creating metadata models that can facilitate correlation. An example to better understand the topic is the correlation between gene mutations and a tumour progression index, as could be an imaging biomarker [47].

#### Diversity of ontologies

Besides the diversity of data, there is also a wide variety in the models built for homogenising and storing these data, leading to ontology “silos.” As such, ontologies and data models are usually developed for describing limited sets of data and cannot scale when other types of data need to be stored using the same model. More than this, various groups are performing extensions to ontologies that are not synchronised and compatible with each other, thus leading to several variations of the same ontology, which complicates the ontology selection and its reuse. Currently, no single ontology is sufficient, and usually, multiple ones have to be combined to fully perform project-specific data integration and homogenisation, as usually the needs of each project differ from the exact ones that the existing models try to cover.

#### Data harmonisation

The variation of multicentre data is caused by the heterogeneity of acquisition equipment, which may be seen in biomedical signals, computed tomography, MR imaging, and pathology images. Vendor-specific detector systems, coil sensitivity, positional and physiologic fluctuations during acquisition, and magnetic field variations in MR imaging, among other factors, all contribute to this variability. Some radiomics properties are non-reproducible even when utilising a fixed acquisition methodology for multiple scanner manufacturers, according to studies. Berenguer et al. [48], for example, investigated the repeatability of radiomics features on five different scanners using the same acquisition methodology and found significant variances, with 16% to 85% of radiomics features being repeatable. As such, it is obvious that in large-scale digital healthcare research, removing the bias and variation of multicentre data has always been a challenge, requiring the capacity to combine clinical characteristics retrieved from data gathered by diverse scanners and protocols, to increase stability and robustness. Data harmonisation, in particular computational data harmonisation, provides an effective solution for analysing multicentre and multiscanner acquired medical imaging data along with metadata. By changing data formats, terminologies, and measurement units, data harmonisation refers to merging data from several sources into a single coherent data set. It is mostly used to resolve difficulties produced by non-identical annotations or records between operators or imaging systems, when downstream clinical tasks necessitate the usage of a consistent methodology. Besides, the harmonisation of data collected from data providers participating in the research and existing data from open databases is a necessary step for their use in data-driven research. The harmonisation process includes the evaluation and management of the compatibility of data acquired by various sites and heterogeneous sources [49].

In this scope, a number of existing harmonisation methods exist [50]. As far as DICOM metadata, curation workflows have been defined for all DICOM-defined objects. The Perl Open Source DICOM Archive (POSDA) incorporates DICOM validation rules and guides de-identification processes, including validation and correction of linkages, inconsistencies at DICOM series/study/patient level, encoding errors, and more [51]. Other tools are mentioned in [52]. With respect to automating data analysis, an extract-transform-load (ETL) procedure has been proposed by Godinho et al. [53], as the Rule-Based Data Cleansing, based on the Dicoogle [54], an open source PACS archive. However, a more generic and standard-based solution, not bound to a PACS system or a legacy system, would be preferable. When it comes to calculated features, harmonisation methods refer to mathematical transformations applied to the features, to account for the different vendors’ raw data, as in the ComBat system [55].

Concerning imaging biomarkers, the issue of standardisation relates to both simple and more complicated biomarkers pertaining to radiomics. Imaging biomarkers refer to features that are relevant to a patient’s diagnosis or prognosis. These biomarkers are usually extracted through calculating image intensities or distributions, given by machine learning and mathematical modeling algorithms. For instance, the GLCM can be used as an independent prognostic factor in patients with surgically treated rectal cancer [56]. However, while the IBSI initiative standardises which data to extract, technical and human segmentation parameters may still induce variability in such outputs. For example, images acquired with different acquisition protocols alter the absolute values of biomarkers without reflecting any actual biological variance. This can lead to the weak reproducibility of quantitative biomarkers and limit the time-series studies based on multi-source datasets. Data harmonisation is a solution that can be adapted to both images and image features to eliminate the non-biological variances.

#### FAIR principles

Although FAIR principles do not necessarily require harmonisation, current practices for health data management, with respect to data reuse for research and new knowledge extraction, suggest data *FAIRification* as part of the integration and harmonisation of multisite health data [57]. FAIR principles provide guidelines to improve the Findability, Accessibility, Interoperability and Reuse of digital assets. *Findability* is related to making the data easy to find for both computers and humans, by providing appropriate, rich, clear, and unambiguous metadata for automatic discovery. *Accessibility* has to do with the ways that data can be accessed using a standardised, open, free and universally implementable communication protocol. *Interoperability* on the other hand has to do with using an appropriate language for knowledge representation, the exploitation of standard vocabularies enabling the data to interoperate with applications, or workflows for analysis, storage, and processing. *Reuse* has to do with optimising the reuse of data. To achieve this, metadata and data should be well-described so that they can be replicated and/or combined in different settings. This procedure includes as crucial steps the semantic definition, the definition of access rights coupled with data de-identification and pseudonymisation, the definition of metadata, the data curation and validation, the data versioning, indexing, and linking. These mainly refer to the raw medical data (imaging data accompanied by non-imaging data: clinical status, laboratory exams, and therapeutic procedures/outcomes), whose secondary use is crucial in research, as well as to derived data, *i.e.*, features produced from raw data with some computational procedure. Of note, regarding data-driven AI research, the role of metadata is crucial in supporting not only the generation of models, but also AI trustworthiness, analysis of bias, etc.

#### Legal framework

Legal, ethical, privacy, and security requirements emerging from, among others, the charter of Fundamental Rights of the European Union [58], the Clinical Trials Regulation [59], the General Data Protection Regulation [60], the World Medical Association Declaration of Helsinki [61], are essential prerequisites when harmonising sensitive data for developing AI for disease management and research purposes. Health data are obviously sensitive data and as such each integration/harmonisation approach should be done on the basis of the following principles: data minimization (including anonymisation and pseudonymisation) and accuracy; informed consent, lawfulness and further processing of personal data; transparency and communication objectives; privacy data protection by design and default; continuous risk assessment; data security (integrity and confidentiality) and storage limitation; patient’s rights and data subject’s rights; anonymised collection of essential personal data; accountability for data processing; data ownership and intellectual property rights.

All these principles should be met by any harmonisation approach, which in many cases can make the whole process more difficult, however always ensuring that the legal and ethical framework is respected.

### The approach of European Union projects focusing on health imaging

In this section we report on the strategies and the metadata models adopted by the five projects of the Artificial Intelligence In Health Imaging (AI4HI) network, trying to identify common elements and workflows. The main information about these projects is summarised in Table 2. In addition, Table 3 presents the key points on the metadata management of the AI4HI projects.

**Table 2:** Summary of the AI4HI projects, listing their goals, use-cases, types of metadata identified so far

Project	Goal	Considered use cases	Types of metadata	Adopted models
PRIMAGE	To build an imaging biobank for the training and validation of machine learning and multiscale simulation algorithms	Paediatric neuroblastoma and diffuse intrinsic pontine glioma	DICOM tags Image analysis metadata (registration, denoising, radiomics) Clinical variables	DICOM-MIABIS OMOP CDM
EuCanImage	To build a European cancer imaging platform for enhanced AI in oncology	Eight use cases regarding liver, breast, and colorectal cancer	Imaging data Clinical variables	DICOM-MIABIS ICGC-ARGO
INCISIVE	To improve cancer diagnosis and prediction with AI and big data	Lung, breast, colorectal, and prostate cancer	Imaging data Clinical and biological data	FHIR
CHAIMELEON	To develop a structured repository of health images and related clinical and molecular data	Lung, breast, prostate, and colorectal cancer	Imaging data Clinical variables	DICOM-MIABIS OMOP CDM
ProCancer-I	To develop an AI Platform integrating imaging data and models	Prostate cancer	Imaging data Clinical variables	DICOM-Radiation therapy OMOP CDM with Oncology Extension

*AI* Artificial intelligence, *AI4HI* Artificial Intelligence for Health Imaging, *DICOM* Digital Imaging and Communications in Medicine, *FHIR* Fast Healthcare Interoperability Resources, *ICGC-ARGO* International Cancer Genome Consortium-Accelerating Research in Genomic Oncology, *MIABIS* Minimum Information About BIobank data Sharing, *OMOP CDM* Observational Medical Outcomes Partnership Common Data Model, *SEDI* Semantic DICOM

**Table 3:** Metadata management approaches of the AI4HI projects

Project	Metadata collection	Metadata types	Models used	Unique characteristics
PRIMAGE	Structured e-forms	Imaging, clinical, image radiomic analysis	DICOM for imaging metadata MIABIS for biological samples and tissue OMOP-CDM for clinical	Integration of the DICOM and MIABIS standards, and metadata model that captures the biomechanical/signalling behaviour of tumours
EuCanImage	Structured e-forms	Imaging, clinical	DICOM-MIABIS for imaging data Extension of ICGC-ARGO for clinical variables	Link between imaging and non-imaging data
INCISIVE	Structured e-forms	Clinical, biological, imaging	Multiple terminologies for clinical data (*e.g*., SNOMED-CT, ICD10, ATC classification) FHIR for communication	Data Integration Quality Check Tool employed to identify whether data follow the harmonisation requirements defined
CHAIMELEON	Structured e-forms	Imaging, clinical	DICOM for imaging metadata MIABIS for biological samples and tissue OMOP-CDM for clinical	A multimodal analytical data engine will facilitate interpretation, extraction, data harmonisation, and exploitation of the stored information. The CHAIMELEON repository will ensure the usability and performance of the repository as a tool fostering AI experimentation
ProCancer-I	Data upload tool (e-forms)	Imaging, clinical	DICOM-Radiation therapy for imaging data OMOP-CDM for clinical data	Provides an extension to OMOP-CDM going beyond radiology/oncology extensions and introduces another model (AI passport) for modeling analysis workflows and AI development

*AI* Artificial intelligence, *AI4HI* Artificial Intelligence for Health Imaging, *ATC* Anatomical Therapeutic Chemical Classification (World Health Organization), *DICOM* Digital Imaging and Communications in Medicine, *FHIR* Fast Healthcare Interoperability Resources, *ICD 10* International Classification of Diseases 10, ICGC-ARGO International Cancer Genome Consortium-Accelerating Research in Genomic Oncology, *MIABIS* Minimum Information About BIobank data Sharing, *OMOP CDM* Observational Medical Outcomes Partnership Common Data Model, *SEDI* Semantic DICOM, *SNOMED-CT* Systematized Nomenclature of Medicine Clinical Terms

#### PRIMAGE: PRedictive In-silico Multiscale Analytics to support cancer personalized diaGnosis and prognosis, Empowered by imaging biomarkers

PRIMAGE is a Horizon 2020 funded project (grant agreement number 826494) aimed at building an imaging biobank of two types of paediatric tumours: neuroblastoma and diffuse intrinsic pontine glioma [3, 4]. The project is constructed as an observational *in silico* study involving high-quality anonymised datasets (imaging, clinical, molecular, and genetics) for the training and validation of machine learning and multiscale simulation algorithms.

In PRIMAGE data repositories, for each patient imaging data is linked to their available pseudonymised biological, pathological, and genetics data. All metadata of the PRIMAGE platform are grouped into a so-called “e-form”, which represents the multiomics data collection interface. The fully web-based PRIMAGE platform allows the centralised management of medical images and their analysis through the extraction of imaging biomarkers and the development of multi-scale models. Therefore, we can identify three categories of metadata that are relevant for this platform: image metadata, clinical variables, and metadata relative to the image radiomics analysis. The DICOM-MIABIS model described earlier was developed within the PRIMAGE framework to facilitate linkage and harmonisation of these three types of data inside the platform, as well as to allow the link of the PRIMAGE metadata with other types of biorepositories. The choice of unifying the two standards, DICOM for imaging metadata and MIABIS for biological samples and tissue, was mainly led by the ESR long-term goal of creating a network of imaging biobanks integrated with the already-existing biobanking network [19]. In addition, work is ongoing to map the clinical variables collected in the platform for the two types of paediatric tumours to the OMOP CDM to ensure their harmonisation.

An interesting research development in PRIMAGE is represented by the metadata model that captures the biomechanical/signalling behaviour of tumours. A multiscale patient-specific model has been proposed to predict the spatiotemporal evolution of the tumour after simulating the individualised clinical treatment. The multiscale approach has allowed the integration of various length scales from molecules to whole tumours on different time scales. Starting from the image geometry of the tumour, a macroscopic Finite Element model reproducing the exact tumour geometry was created. In addition, image biomarkers from the patient (DCE-MR imaging maps) are also being integrated into the same personalised Finite Element model, taking into account heterogeneous spatial distribution of cellularity and vascularisation. Both tumours, neuroblastoma, and diffuse intrinsic pontine glioma are characterised by high heterogeneity. In particular, the ANSYS commercial Finite Element software is used. This macroscopic biomechanics finite element-based model allows the evaluation of the non-uniform growth and the residual stresses characteristics of tumours [62]. This macroscopic approach is fed by a multicellular model that regulates the spatiotemporal evolution of the tumour. In PRIMAGE, the critical behaviours of cells within the tumour are captured using a hybrid model, where individual cells are represented by equivalent virtual entities known as “software agents.” The latter are embedded in virtual lattice (a “continuous automaton”) which represents the distribution of non-cellular material in the microenvironment and interacts physically *via* a cell-centred method of displacement resulting from repulsive forces, as described [63]. Cell agents, which represent different cell types are iteratively updated and permitted to divide, differentiate, or die according to rules relating to their current internal state (representing the mutation profile/activation/expression level of a subset of key proteins including MYCN, Alk, TERT), and signals from the local microenvironment (local cell density, oxygen level, presence of particular chemotherapeutic drugs). The implementation of this multiscale strategy is computationally intensive and is currently intractable for whole-tumour scale simulations. For this reason, a particularisation approach has been adopted, whereas 20 elements of the organ scale simulation have been selected for cellular scale modeling at each time step. Cellular scale models are then initiated and executed for a period representing 14 days using the parallelised FLAMEGPU framework, which permits the simulation of millions of cells in tractable timescales [64, 65].

#### EuCanImage: towards a European cancer imaging platform for enhanced AI in oncology

EuCanImage is a Horizon 2020 project (grant agreement number 952103) that aims to build a federated large-scale European cancer imaging platform, with capabilities that will allow the development of multi-scale AI solutions that integrate clinical predictors into dense, patient-specific cancer fingerprints [5]. From a clinical perspective, EuCanImage is divided into eight use cases, tackling liver, breast, and colorectal cancer types. For each individual in each use case, there are a series of MR images of the tumour and around 80 non-imaging parameters ranging from age at diagnosis and gender to information about treatment, comorbidities, etc. As many hospitals and clinical centres participate in this project, the source and format of these data are highly heterogeneous.

**Fig. 2:**
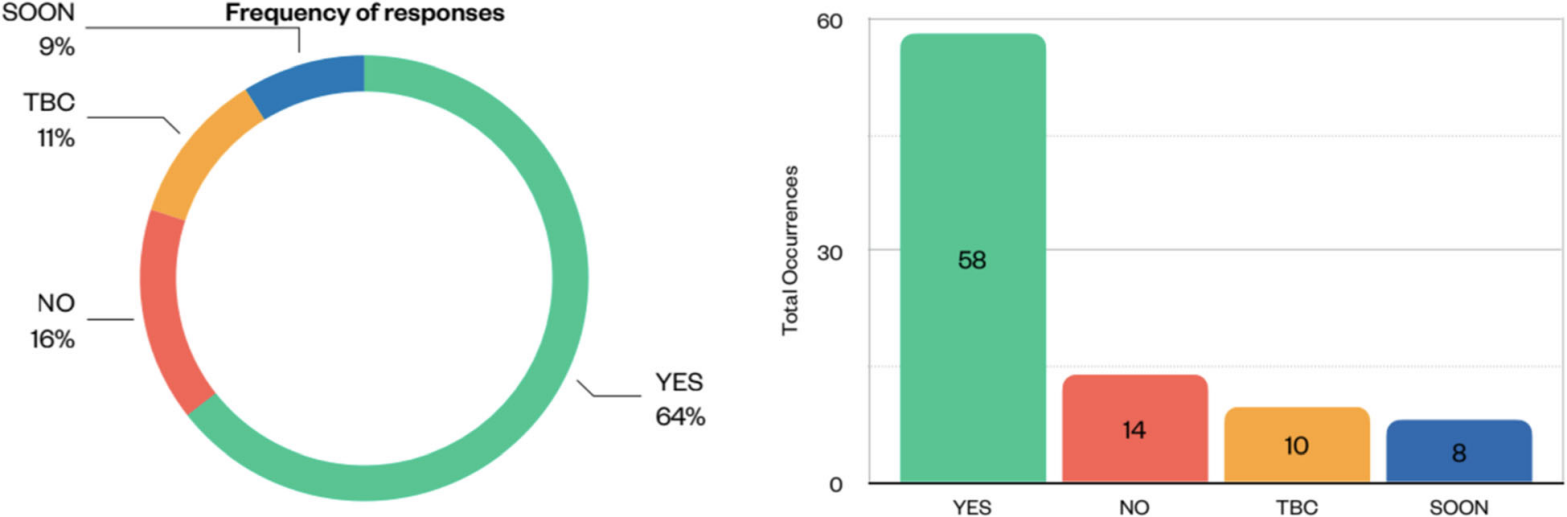
Level of EuCanImage variable mapping into the Accelerating Research in Genomic Oncology (ARGO) model based on the clinical use cases, at the time of assessment (July 2021). *TBC* To be confirmed

To deal with heterogeneous sources of imaging data, the data model for imaging data will be based on the integrated DICOM-MIABIS structure described before, because it features tables and attributes to describe image metadata that are particularly suited to EuCanImage’s needs. The ICGC-ARGO dictionary was selected as a basis for the EuCanImage data model of clinical variables, because it is used to analyse data in the ICGC platform, which contains specimens from 100,000 cancer patients with high-quality clinical data. In addition, several funded projects are using the model, including EuCanCan [66], an Horizon 2020 project in cancer research in Canada and Europe. A dedicated EuCanImage working group, including the ICGC model curator, reviewed all data types/variables individually based on the eight clinical use cases that are part of the EuCanImage project. The extent to which the parameters can be mapped onto the ARGO dictionary was assessed, the potential gaps were identified based on feedback from the clinical partners and clinical data, and the ARGO schema was extended accordingly to obtain a comprehensive data model taking into account heterogeneity between sites. From this qualitative analysis, descriptive statistics reflecting the proportion of variables that are already represented in the ARGO dictionary was derived (Fig. 2). Based on the eight clinical use cases (liver, colorectal, and breast tumours), the dictionary already has implemented 64% of the parameters, ranging from 44 to 80% across use cases. The ARGO dictionary is being extended to account for an additional 9% of the missing parameters, while other parameters are currently under discussion.

#### CHAIMELEON: accelerating the lab to market transition of AI tools for cancer management

The CHAIMELEON project (grant agreement number 952172) aims to develop a structured repository of health images and related clinical and molecular data on the most prevalent cancers in Europe: lung, breast, prostate, and colorectal [6]. The key objectives of CHAIMELEON are to establish a European Union-wide interoperable repository with quality-checked imaging data as a resource for developing and testing AI tools for cancer management; to set up a distributed infrastructure building on existing initiatives; to ensure the sustainability of the repository beyond the project runtime; and to develop novel data harmonisation technologies for handling multicentre, multimodal, and multiscanner data. The project involves the collection of images of over 40,000 patients but also has the ambition to include clinical data associated with the images. In order to represent such data within the repository, the strategy already tested in the PRIMAGE project, of which many CHAIMELEON researchers are partners, will be used. A specific CHAIMELEON work package is dedicated to the sustainability of the biobank. The work package foresees that the imaging biomarkers, which will be developed within the biobank, are correctly represented and encoded to be linked to the non-imaging data.

Data acquired at multiple centres with different scanners (cross-vendor/cross-institution image datasets) will be used to access a vast amount of health imaging datasets. Due to a lack of consistency of source medical images generated from different equipment vendors, models, and releases, as well as the lack of an appropriate framework in terms of image acquisition/reconstruction, the quantitative image features and parameters values and ranges extracted from images acquired at one centre may not be reproducible from images acquired at another centre.

In the context of secondary use of health imaging data, the reproducibility of quantitative imaging biomarkers in radiomics is critical. One of the major aims of CHAIMELEON project is to contribute to imaging data harmonisation. Various harmonisation approaches based on image preprocessing and postprocessing will be proposed, including a disruptive approach based on the use of AI models to generate synthetic images adjusted to a common harmonisation framework, harmonising the quantitative imaging biomarkers results, and ensuring that the authenticity and integrity of each synthetic coherent image is properly secured.

#### INCISIVE: a multimodal AI-based toolbox and an interoperable health imaging repository for the empowerment of imaging analysis related to the diagnosis, prediction, and follow-up of cancer

The INCISIVE project [8] is a Horizon 2020 funded project (grant agreement number 826494) focusing on improving cancer diagnosis and prediction with AI and big data. Its aims are to develop, deploy, and validate: (a) an AI-based toolbox that enhances the accuracy, specificity, sensitivity, interpretability, and cost-effectiveness of existing cancer imaging methods; and (b) an interoperable pan-European federated repository of medical images that enables secure donation and sharing of data in compliance with ethical, legal, and privacy requirements. The long-term vision of INCISIVE is, by increasing accessibility and enabling experimentation of AI-based solutions, to showcase its impact, towards their large-scale adoption in cancer diagnosis, prediction, treatment, and follow-up.

Four important cancer types are considered (lung, breast, colorectal, and prostate cancer), and different challenges are recognised in each cancer type, seeking leverage *via* data-driven AI solutions, based on imaging and other clinical and biological data. Retrospective and prospective studies are set up in five countries (Cyprus, Greece, Italy, Serbia, and Spain), to collect and share a multitude of data towards enabling both the AI toolbox and the federated repository. These data are divided into two categories: (a) clinical and biological data; and (b) imaging data. The first category, provided in structured text form, includes demographic and medical history data, histological and blood markers, treatment and tumour details, as well as the imaging acquisition protocol. The second category includes body scans in different modalities DICOM format and histopathological images in png or tiff format. These data include distinct time points during the patients’ treatment: (1) diagnosis; (2) after first treatment (surgery or therapy); (3) first follow-up; (4) second follow-up.

**Fig. 3:**
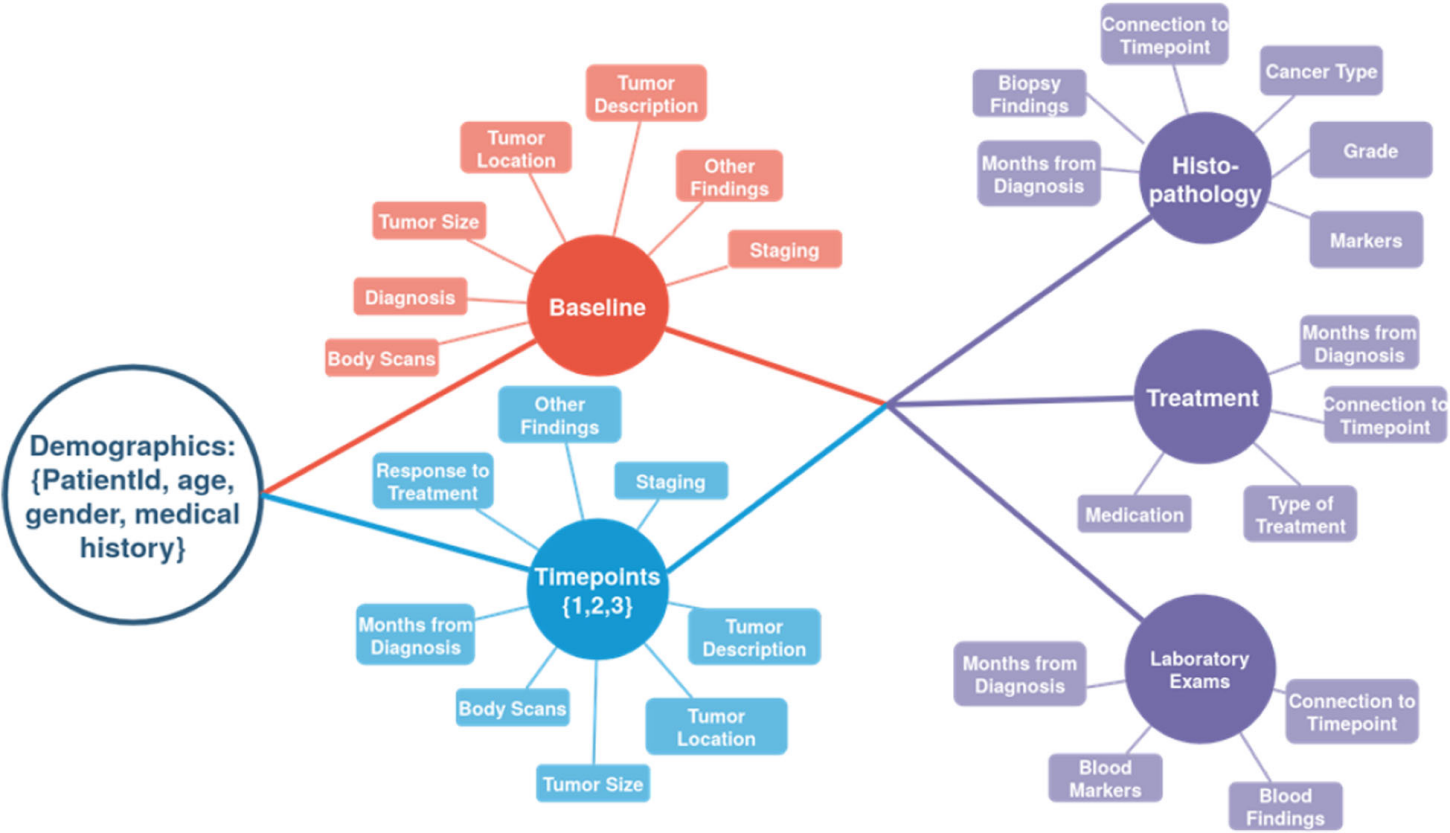
The design of the INCISIVE data model

To construct a model for storing the non-imaging data a template per cancer type was formulated along with the experts, standardising the used fields and adopting terminologies based on medical standards such as the International Classification of Diseases 10 [40] and the Anatomical Therapeutic Chemical (ATC) classification [67]. This structure, presented in Fig. 3, was the basis for the formation of the INCISIVE data model. To that end, the fields of the structured templates were linked to standardised terminologies using the SNOMED-CT vocabulary. To ensure interoperability, an FHIR-based model was created for the communication between the various components of the INCISIVE infrastructure. The data are classified in 3 levels: (i) demographics, patient’s personal information, and medical history; (ii) timepoints, including the baseline and follow-ups, and for each one of them, tumour characteristics resulting from scan examinations, the progression, and status of the disease linked with the actual scan examinations; and (iii) information about histopathology findings, treatment, and blood tests connected to each timepoint.

With regard to the imaging data, the integration procedure included as a first step the analysis of the imaging data from all the sites. The metadata of all DICOM files were processed to investigate harmonisation and de-identification issues and a list with all the attributes for each data provider was created. Furthermore, the same procedure was applied in open datasets and compared with the mock-ups to conclude in an anonymisation standard. Additional attributes related to the image, such as *field of view* and *slice thickness* was also analysed for harmonisation purposes. Eventually, the protocol and data collection procedure for a harmonised data storage was defined. After the data collection and the images de-identification step, which is implemented via the CTP DICOM Anonymizer [68] and a configuration following the DICOM PS3.15 [69] standard, and before data uploading to the repository, a quality check takes place at the local level using Data Integration Quality Check Tool [70], a rule-based engine, implementing domain knowledge, and aims to identify whether data follow the data harmonisation requirements defined within the project, as well as the integrity and consistency of the data.

#### ProCancer-I: an AI platform integrating imaging data and models, supporting precision care through prostate cancer continuum

ProCancer-I (Horizon 2020 grant agreement number 952159) aspires to develop an AI platform integrating imaging data and models, supporting precision care through prostate cancer continuum [9]. The ProCAncer-I project brings together 20 partners, including prostate cancer centres of reference, world leaders in AI and innovative small and medium-sized enterprises, with recognised expertise in their respective domains, with the objective to design, develop and sustain a cloud-based, secure European image infrastructure with tools and services for data handling. The platform will host the largest collection of prostate cancer multiparametric MR imaging, anonymised image data worldwide (> 17,000 cases), based on data donorship, in line with European Union General Data Protection Regulation. Exploiting the available data, robust AI models will be developed, based on novel ensemble learning methodologies, leading to vendor-specific and -neutral AI models for addressing eight prostate cancer clinical scenarios.

The data that will be collected through the lifetime of the project are imaging data and clinical data. The imaging data will be prostate multiparametric MR imaging data and histopathologic (whole-slide pathology images). The clinical data include clinical, prostate specific antigen, prostate specific antigen density, Gleason group, the status of resection margins, presence of extraprostatic invasion, nodal status, post-prostatectomy prostate specific antigen, nodal status, follow-up measurements of prostate specific antigen, toxicity, and quality of life.

Imaging data adopt DICOM-Radiation Therapy and are accompanied with the relevant metadata for capturing the related information. All imaging metadata are currently stored in a metadata catalog, developed explicitly for this purpose. The metadata catalog adopts the OMOP-CDM v6.0 model. However, as the model has limited support for oncological data, the Oncology CDM Extension of the OMOP-CDM is also used for representing the prostate cancer data at the levels of granularity and abstraction required by the project. For radiology exams, although those can be currently registered using the OMOP-CDM, the model does not enable the storage of the subsequent curation process. As such, the ProCancer-I has already introduced a custom radiology extension and is currently working on it in collaboration with the OHDSI Medical Imaging Working Group, focusing on including annotation, segmentation, and curation data as radiomics features that need to be stored as well. Terms found in the source data are also mapped to concepts in the OMOP standard vocabularies to achieve semantic interoperability, whereas in the case that such a mapping cannot be made, non-standard concepts are introduced by the ProCancer-I project.

## Conclusions and future directions

Developing high-quality AI models for health imaging requires access to large amounts of imaging data along with their metadata. However, those datasets might have been produced by different vendors and workflows and use different terminologies and data models to be represented. Many different common data models, ontologies, and terminologies have been developed in order to enable homogeneous representation of the available data. However, despite the plethora of models, typically the specific requirements set by each individual project necessitate the use of multiple models and terminologies in order to appropriately describe the available data. And even that is usually not enough, as extensions are also often required. Recent projects participating in the AI4HI network adopt mostly DICOM-MIABIS structures, the OMOP-CDM along with its extensions, and ICGC-ARGO for modeling imaging and clinical data along with relevant clinical terminologies.

Experiences from all projects should guide future developments in the aforementioned models. For example, already the projects adopting OMOP-CDM joined forces with the Radiology OHDSI working group in order to promote extensions that cover not only the basic radiology information, but also information required for tracking the various curation steps, and for AI subsequent development. The authors believe that *standardisation* is the road to go. However, this is a long and time-consuming process. On the other hand, it is common that different groups might have different interests, and as such *modular, well-defined, and properly described standards* are essential so that the appropriate modules can be selected by the appropriate group according to the specific needs. The adoption of common such standards will enable the easier integration and harmonisation of the collected datasets.

Direct application of one data harmonisation method from one project to another may not be straightforward. This is because different data resources (*e.g*., different scanning modalities) and different clinical questions may require specialised design of the data harmonisation. For example, data harmonisation of tabular data could be different from that of imaging data. To address this issue, transfer learning could be used to enhance the robustness of data harmonisation models by holding a priori knowledge on the way data can vary, and the successful application in-between projects may also be determined by extra training samples in a different project to reduce the uncertainty with respect to the variability of data that models can cope with. However, there is growing evidence that integrating data harmonisation with AI methods allows for robust and accurate predictions on multicentre datasets.

Effectively integrating all these datasets beyond individual project boundaries by specifying a common data model will facilitate the establishment of a common data model for oncology, paving the way for a patient-centric, federated, multi-source, and interoperable data-sharing ecosystem, where healthcare providers, clinical experts, citizens, and researchers contribute, access, and reuse multimodal health data, thereby making a significant contribution to the creation of the European Health Data Space.

However, in that direction, several obstacles should be overcome. At individual project level, the necessary FAIR services should be implemented and be available, enabling regulated, federated access to the data available in each own project infrastructure. The various data sources might use different models for storing data and the corresponding metadata, however exposing a common interface on top for data/metadata access will further promote and ease the integration of the available data. A problem is that most of the projects develop their FAIR services towards the end of the project, when the whole infrastructure is ready, as usually the focus is on the AI tools that each project is delivering and FAIRification is only a minor side project. However, this usually has as a result that no proper time is left for integration with external projects and for promoting such activities. Incorporating FAIR-by-design principles in the first stages of the infrastructure development and producing early in project lifetime relevant services, could boost cooperation opportunities among different projects.

Finally, to ensure a smooth translation from basic science AI research into the clinical arena, explainable AI, XAI, provides a ploy that tries to give rationale, transparency, and traceability of frequently black-box AI algorithms, as well as testability of causal assumptions. In biomedical signal and image processing, especially applications in digital healthcare, determining causation is especially important to justify why a decision is taken and why one intervention or treatment option is preferred over others. Explainable AI is a step toward realising the FATE (Fairness, Accountability, Transparency, and Ethics) and FAIR (Findable, Accessible, Interoperable, Reusable) principles.

## Data Availability

Not applicable.

## Abbreviations

AI: Artificial intelligence
AI4HI: Artificial Intelligence for Health Imaging
CDE: Common Data Element
DICOM: Digital Imaging and Communications in Medicine
EIBALL: European Imaging Biomarkers Alliance
ESR: European Society of Radiology
ETL: Extract-transform-load
FAIR: Findability, Accessibility, Interoperability, and Reuse
FHIR: Fast Healthcare Interoperability Resources
GLCM: Grey level co-occurrence matrix
HL7: Health Level Seven International
IBSI: Image Biomarker Standardization Initiative
ICGC-ARGO: International Cancer Genome Consortium-Accelerating Research in Genomic Oncology
MIABIS: Minimum Information About BIobank data Sharing
MR: Magnetic resonance
OHDSI: Observational Health Data Sciences and Informatics
OMOP CDM: Observational Medical Outcomes Partnership Common Data Model
PACS: Picture Archiving and Communication System
POSDA: Perl Open Source DICOM Archive
SEDI: Semantic DICOM
SNOMED-CT: Systematized Nomenclature of Medicine Clinical Terms
